# Monkeypox as a warning to preserve global herd immunities

**DOI:** 10.1080/21505594.2022.2154424

**Published:** 2023-01-05

**Authors:** Daniel Miranda, David Jesse Sanchez

**Affiliations:** Pharmaceutical Sciences Department, Western University of Health Sciences, Pomona, California, USA

**Keywords:** Monkeypox, polio, measles, vaccination, herd immunity, public health, mpox

The declaration of monkeypox (recently renamed to mpox) as a Public Health Emergency of International Concern (PHEIC) by the World Health Organization (WHO) pushed forth a mitigating health response in an effort to control the developing global epidemic [1]. According to the US Centers for Disease Control (CDC), as of November 2022, the 2022 Monkeypox Outbreak has led to almost 80,000 cases, with most of them being reported in locations that have historically not reported monkeypox infections. Locations such as the USA that have historically seen very few cases of monkeypox had a dramatic burden, with about 29,000 cases as of November 2022. The current diminishing of the number of new infections is likely due to the current immunization strategies focused on targeting vaccination of the most at-risk populations and bolstering public awareness of this disease. However, the limited supply of vaccinations coupled with controversy over who should be getting immunized first had led to an arguably contentious mitigation plan of execution, unable to rapidly reconcile this epidemic in the first months since it began. Furthermore, per recent announcement by the WHO, breakthrough infections, where individuals manifest monkeypox even after vaccination, are a strong reminder that limited vaccination will not be sufficient to stop the spread of monkeypox.

Real-world data on the efficacy and safety profiles of the JYNNEOS and ACAM2000 vaccines in the prevention of monkeypox are critical, as both vaccines were primarily developed for protection against smallpox, a related orthopox virus, and have limited data. In comparison to the clinical trials for vaccinations against COVID-19 (Coronavirus Disease 2019), which reflect up to 30,000 participants, one of the main JYNNEOS clinical trials for determining immunogenicity and safety profiles only looked at 3,003 participants [2]. Additionally, most of the vaccine data used to support the implementation of these vaccines are inferred from animal studies [3]. Furthermore, the main vaccine used in the eradication of smallpox, Dryvax, and its replacement, ACAM2000, both have safety profiles that would be a cause for concern if given similar scrutiny as undergone by COVID-19 vaccines.

One important factor suspected in facilitating the current, global spread of monkeypox is the relatively low-level immunity against smallpox that exists in our worldwide population, with more than 70% of the global population being born after the discontinuation of the historically common administration of smallpox vaccines. Following this general and far-reaching discontinuation of smallpox vaccinations, the level of predicted resistance to monkeypox in the population has notably diminished. Modeling of monkeypox outbreaks in the Democratic Republic of the Congo has predicted that as smallpox immunity decreases in a population with a prevalent propensity for exposure to animal reservoirs of monkeypox, there is a greater likelihood of establishing an endemic or human-adapted strain of monkeypox [4]. And indeed, in 2016, epidemiological studies in the Democratic Republic of the Congo yielded an outbreak with increased human-to-human transmission, within households and the community, that poignantly foreshadowed the current outbreak [5].

There is also complementing suggestion that the diminishing poxvirus immunity in the population may be a contributor to the spread of monkeypox as seen in the age distribution of monkeypox-infected individuals. For example, the SHARE-net collaborative showed that cases of monkeypox had an age median of 38 years (range, 18–68 years of age [6]), while the CDC Multinational Monkeypox Response Team showed that cases in the USA, between May and July 2022, had a median age of 35 years (Interquartile range (IQR): 30–41 years of age [7]). This age distribution, identifiably below the age cutoff for the likelihood of smallpox immunization, implies that a lack of smallpox immunity may increase susceptibility and the spread of monkeypox to regions outside of regions endemic to monkeypox.

The prevalence of zoonotic variants, both re-emerging and completely novel viruses entering our society, is an important foretoken that global herd immunity is only as strong as the least protected regions. As a reminder, herd immunity occurs when an appreciable percentage of a population is immune to a particular infectious agent, lowering the spread of that agent and indirectly lowering the probability of a person that is not immune from being infected by that agent [8]. While most of the Western population is being introduced to the idea of monkeypox only this year, the virus has been and continues to be endemic in parts of Africa – only making its way westward in 2003, when the USA had an outbreak due to importation of small animals [9,10]. An editorial by Murphy and Ly additionally discusses the importance of transmission between other species and humans [11]. The increased exposure of the human population to monkeypox, accompanied by a decreased percentage of the population immunized against orthopox viruses, is a textbook epidemiological algorithm ending in a monkeypox outbreak. In hindsight, opportunities to invest in and develop much-needed immunization strategies to fortify public health in endemic regions – and a worldwide prophylactic benefit thereon – were missed.

**Figure 1.**
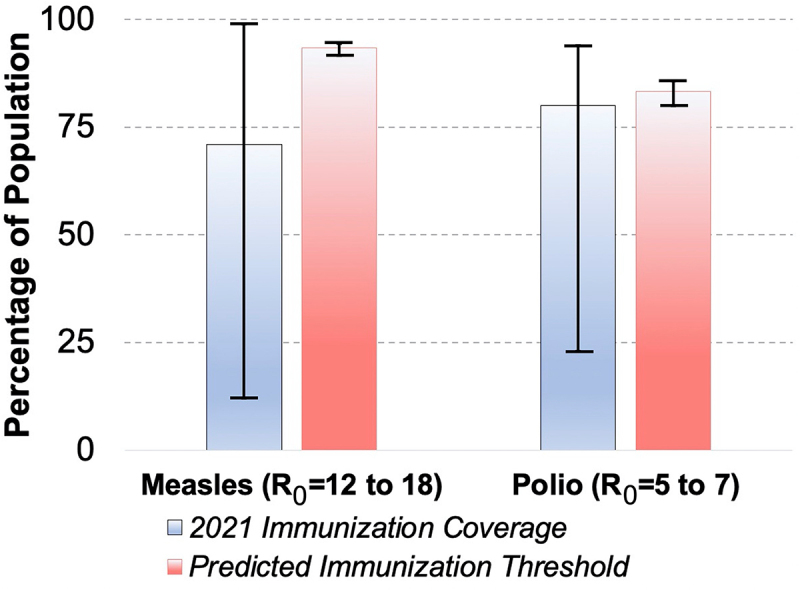
Diminishing Global Immunization Coverage Using data reported by the WHO, global levels of immunization against poliovirus and measles virus are graphed with bar included to show the immunization rate in the countries with both the maximum immunization rate and minimum rate. Using the estimated R_0_, the basic reproduction number of the virus, herd immunity thresholds were calculated with the formula 1 − 1/R_0_ and graphed for both poliovirus and measles with the maximum and minimum predicted. Overall, this shows that different countries fall dangerously outside of the herd immunity thresholds highlighting public intervention targets.

However, the outbreak of monkeypox throughout the world is not the only revealing case of a breakdown in global herd immunity to a viral infection. Just as monkeypox was spreading globally, poliovirus began to surface again with the first case of poliovirus infection reported in the USA since 2005 [12]. Poliovirus has an efficacious vaccine, that is both well understood and has a reputable safety profile. However, while the polio vaccination rate for children aged 24 months in the USA is 92.6% according to the CDC, the local polio vaccination rates in some counties of New York State range from 92% to 54%, with a state average of 79%, according to the New York State Department of Health. A herd immunity threshold is the percentage of a population that needs to be immune to an infectious pathogen to stop its spread [13]. The fact that poliovirus is appearing in a population with an immunization rate below the predicted herd immunity threshold of about 80% for poliovirus, should be a cause for immediate concern. This poliovirus case echoes the outbreaks of measles in the USA in 2019 that continue to smolder as immunization rates hover at, or sometimes below, the herd immunity threshold for measles virus [14]. That measles outbreak is often associated with vaccine refusal and concurrent with cases linked to international travel, but those might not be the only factors – as some have suggested that local, sustained transmission of measles in a population, with immunization rates hovering near the herd immunity threshold may be partially involved [15]. Figure 1 shows the global range of immunization coverage in different countries and the potential for outbreaks of polio and measles and again emphasizing that our global community is only as protected as the areas with the least immunization rates.

These different types of viral outbreaks, albeit having existing and efficacious immunizations, remind us that having the tools to stop outbreaks is different than executing prevention and extinguishing outbreaks. It also underscores that herd immunity for a population requires constant upkeep and vigilance – this herd immunity does not last forever. Most importantly, these situations reinforce the fact that we must remain on-guard against outbreaks of both new and old infections. Monkeypox is a known viral disease, that is endemic in well-populated parts of the world. The idea that eradication may not be permanent, wherein hidden endemics may persist in animal reservoirs or in low-level infections and may soon become the norm in the overall infection cycle. To prevent this, we must focus on blocking these pathogens from establishing themselves in our human population, by utilizing key resources directed toward global prevention and spread. Ultimately, waiting until the waves of infection spread over to Western countries or beyond isolated parts of the region will be too late to suppress an outbreak.
